# A Rare Case of Malignant Pleural Mesothelioma in a Young Healthy Male Without Asbestos Exposure

**DOI:** 10.7759/cureus.17199

**Published:** 2021-08-15

**Authors:** Sohaib Khatib, Osama Asad, Hussein Asad, Taher Sabobeh

**Affiliations:** 1 Internal Medicine, University of Missouri Kansas City School of Medicine, Kansas City, USA; 2 Internal Medicine, Jordan University of Science and Technology, Irbid, JOR; 3 Pulmonary and Critical Care, University of Missouri Kansas City School of Medicine, Kansas City, USA

**Keywords:** mpm, malignant pleural mesothelioma, asbestos, epithelioid mesothelioma, malignant pleural effusion

## Abstract

Malignant pleural mesothelioma (MPM) is a highly aggressive malignant tumor that arises from mesothelial cells of pleural cavity. The main risk factor for MPM is asbestos exposure with most cases discovered in elderly males after a long latency period. However, here we report a rare case of MPM diagnosed in a healthy young male patient without significant asbestos exposure.

We report the case of an otherwise healthy 47-year-old male who presented with one week of exertional dyspnea and chest pain. Chest X-ray showed unilateral large pleural effusion. Chest CT scan revealed confluent right hilar mass and pleural thickening. Pleural fluid analysis showed exudative features. Cytology was negative for malignant cells. Core tissue biopsy showed features of epithelioid mesothelioma.

Although most cases of MPM have been reported in elderly male patients with significant asbestos exposure, more research is needed to explain the pathogenesis of MPM in young patients without asbestos exposure.

## Introduction

Malignant mesothelioma is an aggressive tumor of serosal surfaces such as the pleura and peritoneum that is increasing in frequency around the world [1]. The most common site is the visceral pleura (90%), followed by the peritoneum. The vast majority of mesothelioma is caused by occupational asbestos exposure with predominance in males [2]. The incidence of this malignancy has been increasing for several years parallel with the increase in asbestos use, but with a lag of time of 25-40 years [3].

Two thousand to 3000 patients are diagnosed with malignant pleural mesothelioma (MPM) in the United States yearly. WHO mortality database reporting an overall mortality rate of less than 10 deaths per million population confirms that the disease is indeed rare. However, the age-specific mortality rate increased steeply with age, to exceed 100 deaths per million in elderly males [4]. It is highly aggressive with a median survival of 9-17 months. Women were found to have a more favorable outlook than men [5].

MPM can present clinically as localized pleural tumors or as a diffuse pleural disease with effusion and obliteration of the pleural space. The most common and aggressive variant is diffuse MPM; another less common variant is localized pleural mesothelioma [6]. In this report, we discuss a case of MPM in a young healthy male without a history of asbestos exposure. 

## Case presentation

We report a case of a 47-year-old African-American male with a past medical history significant for alcohol use and tobacco use who presented to the hospital complaining of chest pain and shortness of breath. The patient stated that one week before presentation he was in his usual state of health in which he normally feels well, eats well, and can walk many blocks, and even run without experiencing any chest pain or shortness of breath. One week before presentation to the hospital, the patient started to complain of right-sided chest pain, sharp in nature, 7/10 in severity, worse with exertion, movement, and deep breaths, and improves with lying down. In addition to chest pain, the patient also experienced exertional shortness of breath where he gets easily winded after walking for about 20 feet. However, he denied any shortness of breath at rest.

The review of systems was negative for fever, chills, headache, body aches, hemoptysis, cough, rhinorrhea, nausea, vomiting, abdominal pain, diarrhea, lower-extremity swelling, or dysuria. His social history was significant for smoking cigarettes with 10 pack-years. The patient also reported drinking 4-6 beers daily and occasional hard liquor use. The patient also endorsed occasional marijuana use. The patient reported working as a maintenance man for his entire life, and he reported exposure to black mold in the past; however, he denied any exposure to asbestos, concrete, or heavy metals.

**Figure 1:**
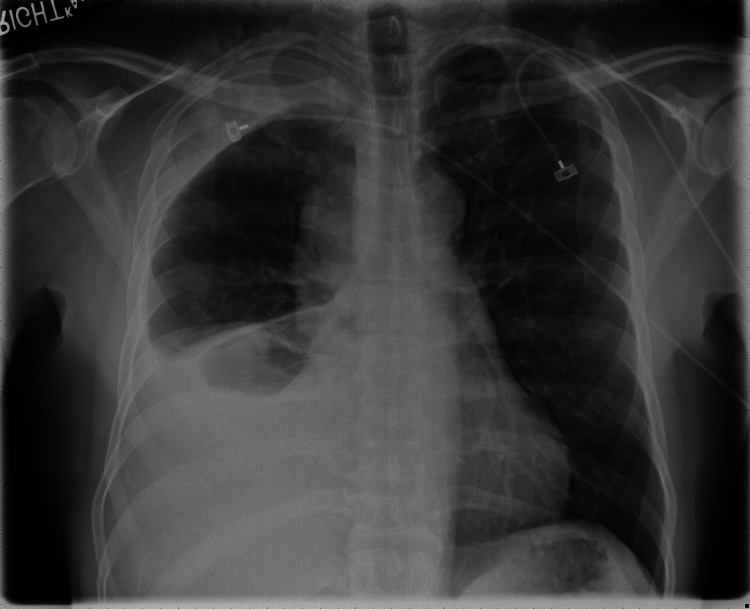
Chest X-ray Chest X-ray showing large unilateral right pleural effusion.

Upon evaluation in our hospital emergency department, his vital signs were stable; the patient was having normal oxygen saturation on room air. Blood workup was only significant for normocytic anemia with hemoglobin 11.7. Chest X-ray (Figure 1) showed a large unilateral right pleural effusion.

**Figure 2:**
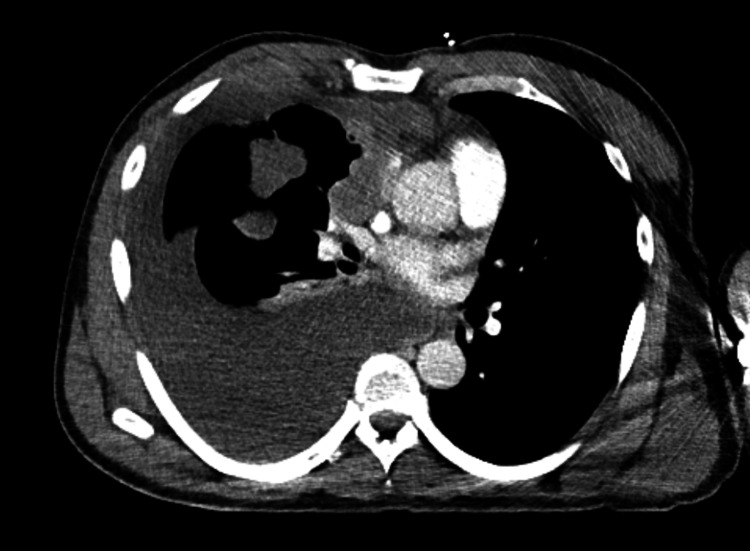
CT chest with contrast, axial view CT chest shows large confluent right hilar mass, mass-like pleural thickening in addition to nodular pleural thickening throughout the right hemithorax. Large right-sided pleural effusion with significant mass-effect and compressive atelectasis on the right lower and middle lung lobes.

Due to concerns of association of unilateral pleural effusion with malignancy, CT chest with contrast was done and it showed a large confluent right hilar mass, mass-like pleural thickening in addition to nodular pleural thickening throughout the right hemithorax. The imaging also showed large right-sided pleural effusion with significant mass-effect and compressive atelectasis on the right lower and middle lung lobes (Figure 2).

Transthoracic echocardiogram showed normal ejection fraction 55%-60%, mild to moderate pulmonic regurgitation, and features of something extracardiac compressing the right atrium.

The patient underwent right-sided thoracentesis, and 3000 cc of serosanguineous fluid were obtained and sent for labs and cytology. On the same day, the patient also underwent CT-guided right upper lobe lung/pleural mass biopsy without complications. Further imaging was done for staging including CT abdomen and pelvis along with MRI brain; those were negative for metastatic disease. Pleural fluid analysis showed exudative features (Table 1). 

**Table 1:** Pleural fluid analysis Pleural fluid analysis with exudative features. WBC, white blood cells; RBC, red blood cells; LDH, lactate dehydrogenase.

Laboratory value	Result	Normal reference range
Pleural fluid appearance	Cloudy	Normal low clear
Pleural fluid color	Orange	Normal low pale yellow
Pleural fluid glucose	66 mg/dL	NA
Pleural fluid WBCs count	1706/cmm	≤1000
Pleural fluid RBCs count	13066/cmm	≤0
Pleural fluid polymorphonuclear cells	9%	≤25%
Pleural fluid LDH	673 units/L	NA
Pleural fluid protein	4.8 g/dL	NA
Pleural fluid pH	6.8	NA
Pleural fluid protein/serum protein ratio	0.67	≤0.5
Pleural fluid LDH/serum pH ratio	4.6	≤0.6

Cytology was negative for malignant cells. After that, a core right upper lobe lung/pleural mass biopsy was done. Immunostains with appropriate controls were performed on paraffin-embedded tissue for pan-cytokeratin, CK7, CK20, CK5/6, TTF-1, P63, CD45, CD68, CD3, CD5, CD20, CD138, MUM1, epithelial membrane antigen (EMA), calretinin, PAX8, MOC31, vimentin, Mart1, SOX10, and P53. Tumor cells were positive for pan-cytokeratin, CK7, CK5/6, P53, calretinin, focally positive for EMA and vimentin, and negative for the other markers (TTF1, P63, CK20, CD20, CD3, CD5, CD138, CD45, Mart1, SOX10, PAX8, CD68, MOC31, and MUM1). These results were consistent with epithelioid mesothelioma.

Immunostains with appropriate controls performed for WT1, D2-40, and BAP1 showed that tumor cells were positive for WT1 and D2-40 and negative for BAP1, supporting the diagnosis of epithelioid mesothelioma.

Based on the clinical history of pleural mass, histology of the core biopsies, and the immunophenotypic profile, a diagnosis of epithelioid mesothelioma was confirmed. 

## Discussion

MPM is a rare and fatal tumor that predominantly affects males (81%). About 80% of patients with MPM reports prior asbestos exposure [7]. The average latency of MPM after asbestos exposure is around 50 years with most cases diagnosed in the sixth through eighth decades. As MPM is not always associated with asbestos exposure and only a small number of individuals with heavy asbestos exposure develop MPM, other several etiologies have been suggested in the literature. It is suggested that genetic predisposition plays important role in MPM development that even unapparent asbestos exposure can end in tumor development [8]. Another proposed mechanism for MPM development without asbestos exposure is exposure to the tumorigenic simian vacuolating virus 40 as this virus gene sequence has been found in more than 50% of epithelial MPM [9]. It is also hypothesized that innovative nanomaterials (nanotubes) may contribute to the development of MPM in some patients [10].

Patients with MPM usually present with progressive shortness of breath secondary to unilateral pleural effusion. Dyspnea is usually associated with non-pleuritic chest pain likely due to significant chest wall invasion. These symptoms are usually insidious and nonspecific, which can delay diagnosis to 3-6 months. Chest radiographs usually show findings of unilateral large pleural effusion in about 90% of patients. In addition to pleural effusion, a CT scan of the chest also can show pleural thickening (92%), thickening of pleural surfaces of the interlunar fissures (86%), and pleural calcifications in 20% of cases [11]. MRI can also help to assess for tumor invasion to the chest wall or diaphragm. Positron emission tomography has the advantage of detecting distant metastasis. Pleural puncture with cytology can be positive in up to 30% of cases with MPM; however, cytological diagnosis sensitivity is limited [12]. Image-guided percutaneous pleural biopsy is highly sensitive (87%) in confirming the diagnosis. Moreover, video-assisted thoracoscopic surgery is 98% sensitive and 100% specific in the diagnosis of MPM [13]. The histological appearance of MPM is variable and challenging, given its histomorphologic similarities to adenocarcinoma [8]. Special immunohistochemical tests (such as vimentin and calretinin) will aid in confirming the diagnosis. 

Our patient had an unusual presentation of MPM as a young male with no prior asbestos exposure. Although MPM has been reported in young patients, those were more frequently females with prior radiation exposure or family history of breast cancer [14]. A limitation in our study is that although the patient denied any previous exposure to asbestos in his life, it is still possible that he had been exposed to asbestos in his work in maintenance. However, even if he was exposed to asbestos, the period of exposure is still too short to cause MPM. The patient in our report had a typical clinical presentation with gradual dyspnea and chest pain although the duration of his symptoms was much shorter than most cases, which usually last for several months prior to diagnosis. The chest imaging with chest X-ray and chest CT scan showed common findings frequently seen in MPM, and these include unilateral large pleural effusion and pleural thickening. Pleural fluid analysis in our case showed exudative features that are typical with malignant pleural effusion [15]. However, cytology was negative for malignant cells. The definitive diagnosis was made with the pathological examination of CT-guided pleural core biopsy showing features of epithelioid mesothelioma, which is the main histological subtype of MPM (60%-80%) [16].

## Conclusions

MPM is a highly aggressive malignant tumor that usually affects old patients with significant asbestos exposure. However, infrequent cases of MPM have been reported in young patients and patients without prior asbestos exposure. Our case is extremely unusual with a healthy young male patient diagnosed with MPM without previous asbestos exposure in his life. More studies are needed to look for the pathogenesis and risk factors associated with the development of MPM in young patients without asbestos exposure as more cases have been reported recently. 
